# Severe Combined Immunodeficiency from a Homozygous DNA Ligase 1 Mutant with Reduced Catalytic Activity but Increased Ligation Fidelity

**DOI:** 10.1007/s10875-024-01754-1

**Published:** 2024-06-19

**Authors:** Huda Alajlan, Vlad-Stefan Raducanu, Yossef Lopez de los Santos, Muhammad Tehseen, Hibah Alruwaili, Amer Al-Mazrou, Reem Mohammad, Monther Al-Alwan, Alfredo De Biasio, Jasmeen S. Merzaban, Hamoud Al-Mousa, Samir M. Hamdan, Anas M. Alazami

**Affiliations:** 1 Translational Genomics, Centre for Genomic Medicine, King Faisal Specialist Hospital & Research Centre, MBC 3, P.O. Box 3354, 11211 Riyadh, Saudi Arabia; 2 Bioscience Program, Division of Biological and Environmental Sciences and Engineering, King Abdullah University of Science and Technology, 23955 Thuwal, Saudi Arabia; 3 Cell Migration and Signaling Laboratory, Bioscience Program, Division of Biological & Environmental Science & Engineering (BESE), King Abdullah University of Science and Technology (KAUST), Thuwal, Saudi Arabia; 4 Cell Therapy and Immunobiology Department, King Faisal Specialist Hospital & Research Centre, Riyadh, Saudi Arabia; 5 Pediatric Allergy & Immunology, Department of Pediatrics, King Faisal Specialist Hospital & Research Centre, MBC 3, P.O. Box 3354, 11211 Riyadh, Saudi Arabia; 6 College of Medicine, Alfaisal University, Riyadh, Saudi Arabia

**Keywords:** LIG1, SCID, autosomal recessive, whole exome sequencing, homozygous, immunophenotyping, 8-Oxoguanine, magnesium, molecular dynamic simulations, residue interaction network

## Abstract

A cell’s ability to survive and to evade cancer is contingent on its ability to retain genomic integrity, which can be seriously compromised when nucleic acid phosphodiester bonds are disrupted. DNA Ligase 1 (LIG1) plays a key role in genome maintenance by sealing single-stranded nicks that are produced during DNA replication and repair. Autosomal recessive mutations in a limited number of individuals have been previously described for this gene. Here we report a homozygous *LIG1* mutation (p.A624T), affecting a universally conserved residue, in a patient presenting with leukopenia, neutropenia, lymphopenia, pan-hypogammaglobulinemia, and diminished in vitro response to mitogen stimulation. Patient fibroblasts expressed normal levels of LIG1 protein but exhibited impaired growth, poor viability, high baseline levels of gamma-H2AX foci, and an enhanced susceptibility to DNA-damaging agents. The mutation reduced LIG1 activity by lowering its affinity for magnesium 2.5-fold. Remarkably, it also increased LIG1 fidelity > 50-fold against 3’ end 8-Oxoguanine mismatches, exhibiting a marked reduction in its ability to process such nicks. This is expected to yield increased ss- and dsDNA breaks. Molecular dynamic simulations, and Residue Interaction Network studies, predicted an allosteric effect for this mutation on the protein loops associated with the LIG1 high-fidelity magnesium, as well as on DNA binding within the adenylation domain. These dual alterations of suppressed activity and enhanced fidelity, arising from a single mutation, underscore the mechanistic picture of how a LIG1 defect can lead to severe immunological disease.

**Supplementary Information:**

The online version contains supplementary material available at 10.1007/s10875-024-01754-1.

## Introduction

The capacity of cells to survive and to evade cancer development depends on their ability to maintain genomic integrity. This can be seriously jeopardized when nucleic acid phosphodiester bonds are disrupted, whether during normal genetic metabolism or through the action of DNA damaging agents such as ionizing radiation. This disruption can take the form of single-stranded breaks (SSBs), such as naturally occurs between Okazaki fragments, or double-stranded breaks (DSBs) such as those generated during meiosis. Extensive molecular mechanisms exist to detect and correct these breaks, which are ultimately resolved in humans by a trio of DNA ligases [1].

Of the three, LIG1 is the most critical ligase for DNA replication, connecting over 50 million Okazaki fragments during every replication cycle [2]. It is also involved in SSB DNA repair in the base excision repair pathway and may also play a poorly-understood role in DSB repair through the alternative end-joining route [3, 4]. Through its C-terminus it fully envelops nicked DNA [5, 6], in a multi-step process that ultimately seals the phosphodiester backbone after consumption of ATP.

Excessive production of this protein is often detected in cancer cells, suggesting that it is required for their survival, and in vitro studies have shown that greater levels of LIG1 expression are associated with enhanced cell proliferation [7]. Serum starvation and cellular differentiation, on the other hand, are associated with decreased expression [8].

In 1992 the first case of hereditary LIG1 impairment was identified in humans. The patient was born underweight and anemic and had growth retardation, developmental delays, and photosensitivity. Recurring ear and chest infections were also present along with hypogammaglobulinemia, indicative of immunodeficiency. The patient died from pneumonia at the age of 19 [9, 10]. The heterozygous mutations identified in this patient included E566K, which rendered the protein's ATP-binding site inactive, and R771W, situated next to a DNA-binding motif which severely hampered protein function [9, 11].

Since then, additional patients with hereditary LIG1 impairment have been reported. Their clinical manifestations include hypogammaglobulinemia, decreased B and T cell counts, and lymphopenia, suggesting that rapidly dividing adaptive immune cells are more sensitive to LIG1 perturbations [12]. DNA substrate experiments demonstrated that these mutant residues either were affected by non-canonical base pairing architecture, such as failure to ligate nicked DNA containing 3’end 8-oxoGuanine (oxoG), or inactive [13]. Recently a severe combined immunodeficiency (SCID) patient was described with Omenn-like features, bearing compound heterozygosity in LIG1 for a missense mutation and a splice site (truncating) mutation [14]. Intriguingly, all reported LIG1 patients have at least one hypomorphic allele that preserves some residual function, suggesting that full loss of function may be incompatible with life.

In this report we describe the genetic, functional and immunological characteristics of a patient with a hitherto-unreported homozygous LIG1 mutation which was identified through next generation sequencing. She presented with recurrent infections and was found to have anemia, leukopenia, neutropenia, lymphopenia and pan-hypogammaglobulinemia. Molecular analysis of the mutant protein indicated not only diminished ligase activity but also heightened fidelity, providing a detailed mechanistic view of how this LIG1 defect contributes to the onset of SCID.

## Methods

All methods are provided in the supplementary section of this article.

## Results

### Clinical History

**Fig. 1:**
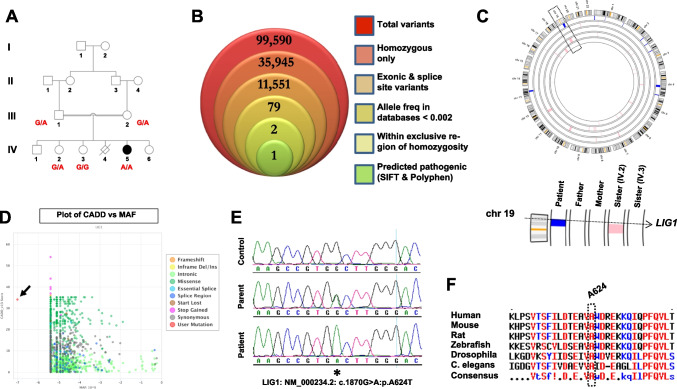
A novel *LIG1* mutation is linked to a severe combined immunodeficiency phenotype. **A** Pedigree of the family in this study, along with genotypes for the individuals who were available for recruitment. **B** WES filtering scheme, shown as a stacked Venn diagram illustrating the total number of variants observed following the addition of each filter. **C** Output from AgileMultiIdeogram, with all regions of homozygosity colored blue (patient) or pink (all other family members) across the genome. Bottom portion shows a close-up of chromosome 19 and the *LIG1* locus. **D** PopViz2 output (shiva.rockefeller.edu/PopViz2/) plotting CADD scores of all known *LIG1* variants against their associated minor allele frequencies. Arrow indicates the A624T mutation. **E** Sequence chromatogram from the patient, alongside a parent and a healthy control for comparison. Asterisk denotes the site of mutation. **F** Protein sequence alignment indicates that A624 is universally conserved across species down to worm

The patient is a product of 32 week gestation, delivered by Cesarean section due to intrauterine growth retardation (IUGR), with a birth weight of 2.3 kg. She was admitted to a local hospital at the age of 2 and 3 months with recurrent chest infections and anemia. At 4 months, the patient developed perianal abscess associated with neutropenia and localized BCGitis. Wound culture grew *Proteus mirabilis* and *Citrobacter freundii*. The parents are 1st degree cousins, and the patient has one healthy brother and three healthy sisters, with a history of previous neonatal death (a preterm 28 week newborn with multiple congenital anomalies and non-immune hydrops), but no family history suggestive of immunodeficiency (Fig. 1A).

The patient was admitted to the hospital at 5 months of age. She was underweight (3.95 kg) and microcephalic with head circumference of 37 cm. She had indurated BCG site with multiple discoloration of the skin. Respiratory examination showed equal bilateral air entry with transmitted sound. Abdomen was soft and lax with no organomegaly.

Immunological work up showed leukopenia, neutropenia and lymphopenia, with counts of 4.87 × 10^9^/L, 0.39 × 10^9^/L, and 1.61 × 10^9^/L, respectively. She had macrocytic anemia, with a hemoglobin of 81 g/L with normal platelets count. She had pan-hypogammaglobulinemia with 0.83 g/L of IgG, less than 0.5 g/L of IgA, and less than 0.25 g/L of IgM. Lymphocyte subsets showed CD3^+^ of 310 /mm^3^, CD4^+^ of 129 /mm^3^, CD8^+^ of 51 /mm^3^, CD19^+^ of 342 /mm^3^, and CD16^+^CD56^+^ of 265 /mm^3^. Percentages of CD3^+^CD45RA^+^ and CD4^+^CD45RA^+^ were 19% and 4%, respectively. Lymphocyte stimulation to phytohaemagglutinin (PHA) was severely depressed (7612 CPM, 6% RR to control).

The patient received hematopoietic stem cell transplantation (HSCT) from her HLA-matched sister without conditioning at the age of 6 months. Her BCG infection was treated with anti TB therapy. She is currently doing well 6 years post-HSCT with 87% lymphoid and 4% myeloid engraftment and with no recurrent infections. Her weight remains low (beneath the 3rd centile). She is maintained on weekly subcutaneous immunoglobulin therapy.

Table 1 provides a summary of the clinical, immunological, and genetic characteristics, as well as HSCT outcomes, of all documented LIG1 cases including the present study.

**Table 1:** Clinical and immunological characteristics, and patient outcomes, of all reported LIG1 deficiencies

	P1	P2	P3	P4	P5	P6	P7	P8 This study
Date of report	1992	2018	2018	2018	2018	2018	2022	2024
Sex	F	M	M	M	M	F	F	F
Onset	2 Years	2 Years	2 Years	2 Months	1 Month	1 Month	Birth	2 months
Age at report	Died at 19 years	19 years	5 years	6 Years	3 Years	6 Years	2.5 years	6 years
Ethnicity	White	White	White	Sudanese	Sudanese	Sudanese	White	Saudi
Infections	Pneumonia, herpes zoster	Diarrhea	Early viral infections	Adenovirus, Rhinovirus, Meta-pneumovirus, Rotavirus, Candidiasis	Rhinovirus, Meta-pneumovirus, RSV	Respiratory infections	Respiratory infection. Rotavirus	Localized BCGitis, Proteus mirabilis, Citrobacter freundii
Anemia	Yes	Yes	Yes	Yes	Yes	Yes	Yes	Yes
Mean corpuscular volume (MCV)	↑	106↑	117↑	105↑	95.6↑	102.1↑	133↑	99↑
Growth	Growth retardation	Normal	Normal	Normal	Normal	Mild retardation	Mild retardation	Microcephaly, growth retadation
Neurological manifestations	Normal mentation	Normal mentation	Normal mentation	Normal mentation	Normal mentation	Normal mentation	Delayed physical and motor	Normal mentation
Organ pathology	Lymphoma	-	-	Multicystic dysplastic kidney	Multicystic dysplastic kidney	Ventricular septal defect	-	-
Complications	Hepatosplenomegaly, Bronchiectasis, Sun hypersensivity	-	-	Severe eczema, severe anemia	Severe anemia	-	Hepatosplenomegaly, Omenn-like skin rash	Anemia
CD3 / mm^3^	240	899	589	730	550	↓	106	310
CD4 / mm^3^	↓	620	271	410	90	No data	87	129
CD8 / mm^3^	↓	170	150	50	20	No data	5	51
CD19 / mm^3^	N	40	229	120	30	↓	107	342
CD56 / mm^3^	No data	50	133	610	930	No data	84	265
γδ T %	No data	↑	40 ↑	19↑	93↑	No data	18.9 ↑	ND
IgG mg/dl	530	70↓	< 2↓	↓	↓	↓	596 (on IV immunoglobulin)	83
IgA mg/dl	↓	8 ↓	115↓	↓	↓	↓	< 7	< 50
IgM mg/dl	N	23↓	36↓	↓	↓	↓	< 4	< 25
PHA	-	-	-	↓	↓	-	↓	↓
Mutation (Zyg)	p.E566K (Het) p.R771W (Het)	p.T415Mfs*10 (Het) p.R641L (Het)	p.T415Mfs*10 (Het) p.R641L (Het)	p.P529L (Hom) p.R771W (Hom)	p.P529L (Hom) p.R771W (Hom)	p.P529L (Hom) p.R771W (Hom)	p.R771G (Het) p.P260* (Het)	p.A624T (Hom)
Immunoglobulin therapy	Yes	Yes	Yes	Yes	Yes	Yes	Yes	Yes
HSCT	-	-	-	Yes	Yes	-	Yes	Yes
Age at transplant	-	-	-	3 years	6 months	-	4 months	6 months
Donor	-	-	-	Match related	Mismatch family donor	-	Matched unrelated	Matched related
Conditioning	-	-	-	Fludarabine, cyclophosphamide, alemtuzumab, anti-CD45	Fludarabine, cyclophosphamide, alemtuzumab	-	Busulfan (reduced dose), Fludarbine, Cyclophosphamide, ATG	No conditioning
Engraftment	-	-	-	100% Lymphoid and myeloid	100% Lymhoid 0% Myeloid 41% B cells	-	90% Lymphoid, 60% Myeloid	87% Lymphoid, 4% Myeloid
Outcome	-	-	-	Alive	Alive, requiring frequent blood transfusion	-	Alive, requiring frequent blood transfusion, poor B cell engraftment on iv immunoglobulin	Alive, poor B cell engraftment, on SC immunoglobulin, Macrocytic anemia (no transfusion required)
References	[9, 10]	[12]	[12]	[12, 20]	[12, 20]	[12]	[14]	

### Identification of a Novel LIG1 Mutation

This family was recruited under an institutional review board-approved protocol, as part of our ongoing effort to identify the genetic causes of rare immunodeficiencies in Saudi Arabia. Due to the consanguineous nature of this family we posited that the underlying mutation was likely present within an autozygous block in the patient’s genome, which would not be shared by any of the other family members. We therefore performed whole exome sequencing (WES) on the patient’s DNA, along with a cataloging of genome-wide genotypes for all family members we had access to (Fig. 1A).

Variants obtained from the WES data were filtered as shown in Fig. 1B, then cross-referenced with the location of all autozygous intervals that were exclusive to the patient (Fig. 1C). This resulted in two variants. One of these was located in *INTS11*, the catalytic subunit of the Integrator complex which is involved in the 3’ processing of small RNA molecules (NM_017871.6:c.1445C > T:p.T482I). Through the use of SIFT and Polyphen-2, two online prediction programs, we eliminated this variant from consideration as both algorithms predicted this change to be non-pathogenic (SIFT: 0.78, tolerated; Polyphen-2: 0.001, benign).

This left us with a single variant, a missense mutation in the *LIG1* gene (NM_000234.2:c.1870G > A:p.A624T) with high pathogenicity prediction scores (SIFT: 0.01, affect protein function; Polyphen-2: 1.0, probably damaging). Plotting its predicted pathogenicity using a third tool, CADD (Combined Annotation Dependent Depletion), revealed a score which was only slightly below that of truncating mutations (Fig. 1D). Sanger sequencing confirmed the mutation and verified its segregation in the family according to the disease state (Fig. 1A, E), while the genotyping data confirmed that the mutation was located within an autozygous stretch that was exclusive to the patient and not shared with the other family members (Fig. 1C, and Fig S1 in Supplementary Material). Protein sequence alignment indicated that the Alanine-624 residue is universally conserved across species down to zebrafish, drosophila and worm (Fig. 1F).

### Immunophenotyping of Patient Cells

**Fig. 2:**
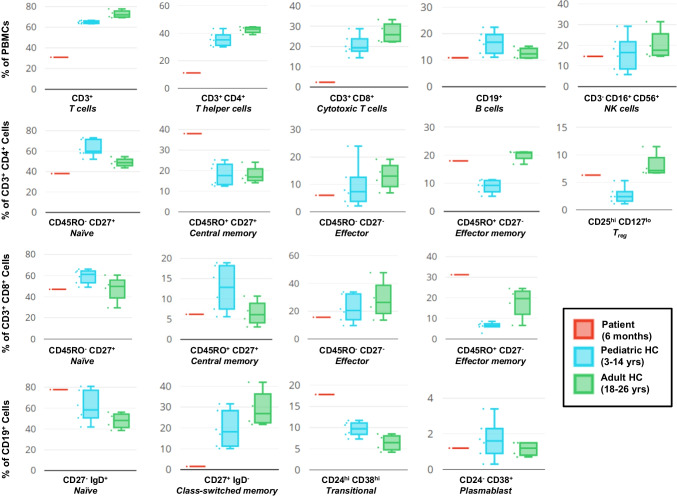
Immunophenotyping of patient PBMCs versus pediatric and adult controls. **A** Investigation of T, B and NK fractions reveals depressed levels of CD3^+^ T cells in the patient, including helper and cytotoxic subsets. **B** Analysis of CD3^+^CD4^+^ and CD3^+^CD8^+^ compartments showing percentages of naïve (CD45RO^−^/CD27^+^), central memory (CD45RO^+^/CD27^+^), effector (CD45RO^−^/CD27^−^) and effector memory (CD45RO^+^/CD27^−^) subsets, as well as regulatory T cells (CD4^+^CD25^hi^CD127^lo^). **C **Assessment of CD19^+^ B cells indicating percentages of naïve (CD27^−^/IgD^+^) and class-switched memory (CD27^+^/IgD^−^) subsets, as well as transitional (CD38^hi^/CD24^hi^) and plasmablast (CD38^+^/CD24^−^) fractions

To functionally characterize the immunological effects of the LIG1 mutation, peripheral blood mononuclear cells (PBMCs) from patient and controls were stained with fluorescent cell-surface antibodies followed by flow cytometric analysis. There was a drastic reduction of CD3^+^, CD4^+^ and CD8^+^ T-cell subtypes, as compared with pediatric and adult controls (Fig. 2). The readings from these PBMCs, sampled at the age of 6 months, are far below the age-matched 10th percentile measurements that have been published for healthy cohorts [15, 16].

Specific analysis of helper T cells (CD3^+^CD4^+^) showed that < 40% of these were naïve. This is considerably below Schatorje and colleagues’ lower limit for this age group. Conversely 18% of CD4^+^ T cells exhibited an effector memory phenotype, whereas the highest observed value in healthy age-matched (5–9 month old) babies was reported at only 1% (Fig. 2) [15]. Our patient’s cytotoxic T cell effector memory percentage was, likewise, well outside the range of our pediatric controls. The central memory helper T subset value lies outside our controls but is within the range provided for our patient's age at sampling.

As with T cells, percent mismatches were also observed with the CD19^+^ B cell population. The proportion of naïve patient B cells was at the top range of our controls. The transitional B cell subset was markedly higher than controls, while the percentage of class-switched memory cells was drastically lower (Fig. 2).

### Patient Cells Reveal Reduced Proliferation and Viability, and Increased Radiosensitivity

**Fig. 3:**
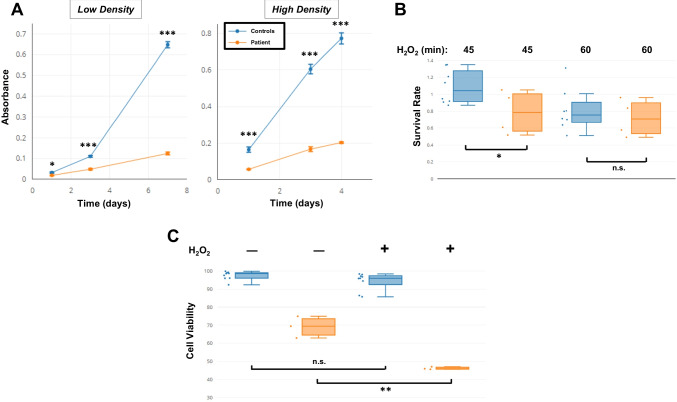
Patient fibroblast cells show diminished proliferation, survival, and viability. **A** Patient and control fibroblast cell lines were seeded at low or high densities, then harvested at various timepoints and analyzed using crystal violet as a proxy for determining growth rates. **B** Cells were tested for their ability to recover following treatment with 200 µM H_2_O_2_ for the indicated time periods, as outlined in the Methods section. Crystal violet readings from day 3 (post-recovery) were normalized to the corresponding day 0 readout. **C** Viability (the DAPI-negative percentage) of patient and control fibroblast cell lines was determined using flow cytometer, in the presence or absence of 200 µM H_2_O_2_ treatment for 30 min. For all experiments a total of 3 control cell lines were utilized. Data are based on three independent experiments for the proliferation and viability assessments, and four for the survival assay. Asterisks denote p-value significance (*p < 0.05, **p < 0.01, ***p < 0.001; unpaired Student’s t-test) and n.s. denotes not significant

To study the impact of the mutant *LIG1* gene at the cellular level, skin biopsy was taken from the patient following her guardian’s informed consent. Fibroblast cells derived from the biopsy were noted to have a significantly slower growth rate than controls, and this was confirmed using crystal violet staining across different seeding densities and timepoints (Fig. 3A). We also checked the effect of H_2_O_2_ on cell recovery by comparing crystal violet (biomass) readout of cells without treatment, to the readout following 3 days of recovery post-treatment. Data indicated a poorer recovery rate for the patient cells for some durations of treatment, but not for others (Fig. 3B). Additionally, flow cytometry analysis revealed a significantly higher percentage of apoptotic cells in patient fibroblasts as compared to controls, as determined by DAPI staining. This trend was clear in the absence of DNA damage induction, and was exacerbated in its presence (Fig. 3C).

**Fig. 4:**
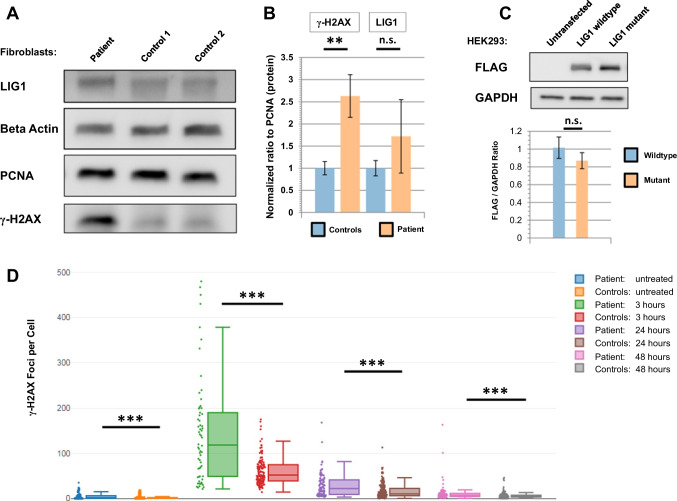
LIG1 patient fibroblasts show enhanced susceptibility to DSB damage. **A** Immunoblot of fibroblast protein lysates, in the absence of any treatment, reveals high basal levels of γ-H2AX staining in the patient. Both beta actin and PCNA were used as loading controls. Image is representative of three independent experiments. **B** Quantification of the immunoblot data, based on normalization of the γ-H2AX readings to PCNA. Normalization to beta actin revealed the same pattern (data not shown). **C** Top: immunoblot of cell lysates from HEK293 cells transfected with FLAG-tagged wildtype and mutant LIG1 expression vectors. Bottom: aggregated data (from 12 transfections) reveals no significant difference in the FLAG-to-GAPDH ratio between the two vectors. Error bars indicate SEMs. **D** Radiation sensitivity assay. The patient and two control fibroblast cell lines were either left untreated or were subjected to 4 Gy ionizing radiation, then allowed to recover for the given time periods. The box plots demonstrate that patient cells had considerably higher γ-H2AX staining at all time periods. For each experiment an average of 45 cells per cell line were scored for every time point, and data are based on two independent experiments. Asterisks indicate significance levels (*p < 0.05, **p < 0.01, ***p < 0.001; unpaired Student’s t-test). Error bars indicate SEMs

We hypothesized that patient cells would be susceptible to buildup of DNA damage, even in the absence of external agents. By immunoblot analysis we observed that patient fibroblasts did indeed exhibit buildup of γ-H2AX, an established marker of the early cellular response to double-stranded DNA breaks (Fig. 4A) [17]. This finding was reproducible and the difference between patient and controls was significant. We also assessed protein levels of LIG1 and observed a non-significant trend of greater expression in the patient cells (Fig. 4B). To investigate if the missense variant was affecting protein stability, whether positively or negatively, human embryonic HEK293 cells were transfected with FLAG-tagged LIG1 plasmid using both the wildtype (WT) and mutant forms. Immunoblot analysis was used for comparing the exogenous LIG1 protein levels (Fig. 4C). The data did not reveal any notable difference between the two groups, suggesting that pathogenesis was not due to any changes in protein stability.

Next, we investigated the effect of another DNA damaging agent by conducting a radiosensitivity assay. Fibroblasts were seeded on poly-L-lysine coated coverslips, then exposed to 4 Gy ionizing radiation and allowed to recover for 3, 24 or 48 h, along with a no radiation control. Probing the cells with fluorescently-labelled anti-gamma-H2AX revealed higher baseline staining in the patient cells, in the absence of treatment (Fig. 4D). This was in line with our immunoblot results. Additionally, at all recovery timepoints the patient cells exhibited significantly higher numbers of gamma-H2AX foci per nucleus, indicating more susceptibility to radiation and a slower rate of DNA damage repair. A previous radiosensitivity study on LIG4-deficient fibroblasts indicated that these, on average, generated three times as many gamma-H2AX foci per nucleus as a wildtype line across all timepoints [18], which is largely analogous to our LIG1 findings. By comparison, fibroblasts deficient in Artemis, another SCID-causing gene with a role in DSB repair, generated substantially fewer foci than the LIG4 mutant cells and were faster in returning to basal levels.

### Cell Cycle Defects in Patient Cells

We noted that growth rates for the patient fibroblast cells were drastically slower than controls, suggesting a cell cycle defect. To examine cell cycle characteristics in the presence or absence of induced DNA damage, cells were assessed as-is or were exposed to 4 Gy ionizing radiation then allowed to recover for up to three days. At various timepoints cells were treated with propidium iodide and observed by flow cytometry to track their cell cycle. At baseline (without treatment), the majority of patient cells were in G1 (Fig. S2A), at a percentage significantly higher than controls, and with significantly fewer cells in S and G2 (Fig. S2B). This reinforced our earlier observation of impaired patient cell growth and suggested G1/S checkpoint difficulties. Following radiation, patient cells were depleted from G1 and began accumulating in G2 at significant levels, and this indicated that the previous buildup in G1 had not been due to increased senescence (G0). Even 72 h post-radiation, the percentage of cells that had effectively exited G2 was low. This contrasted with control cells which exhibited a G2/M spike at 24 h, but then were largely able to progress through mitosis so that by 72 h the percentage of cells in G2 had reverted to basal levels (Fig. S2B).

### A624T Displays Reduced Catalytic Activity

Human LIG1 ligation catalysis and fidelity are controlled by two Mg^2+^ binding sites [19]. The first Mg^2+^ (called catalytic Magnesium) is strictly required for catalysis and provides some basic level of fidelity against a limited number of substrate defects before committing to ligation. The second Mg^2+^ (called high-fidelity Magnesium) causes the enzyme to terminate ligation on substrate defects. A624 is part of a beta sheet (aa. 616–626) involved in the interaction with the catalytic Mg^2+^ ion. Additionally, molecular dynamics simulations (see below) suggest that the effects of the A624T mutation also propagate allosterically to residues involved with the high-fidelity Mg^2+^. These structural considerations prompted us to evaluate the effect of A624T on LIG1 catalytic efficiency and fidelity.

**Fig. 5:**
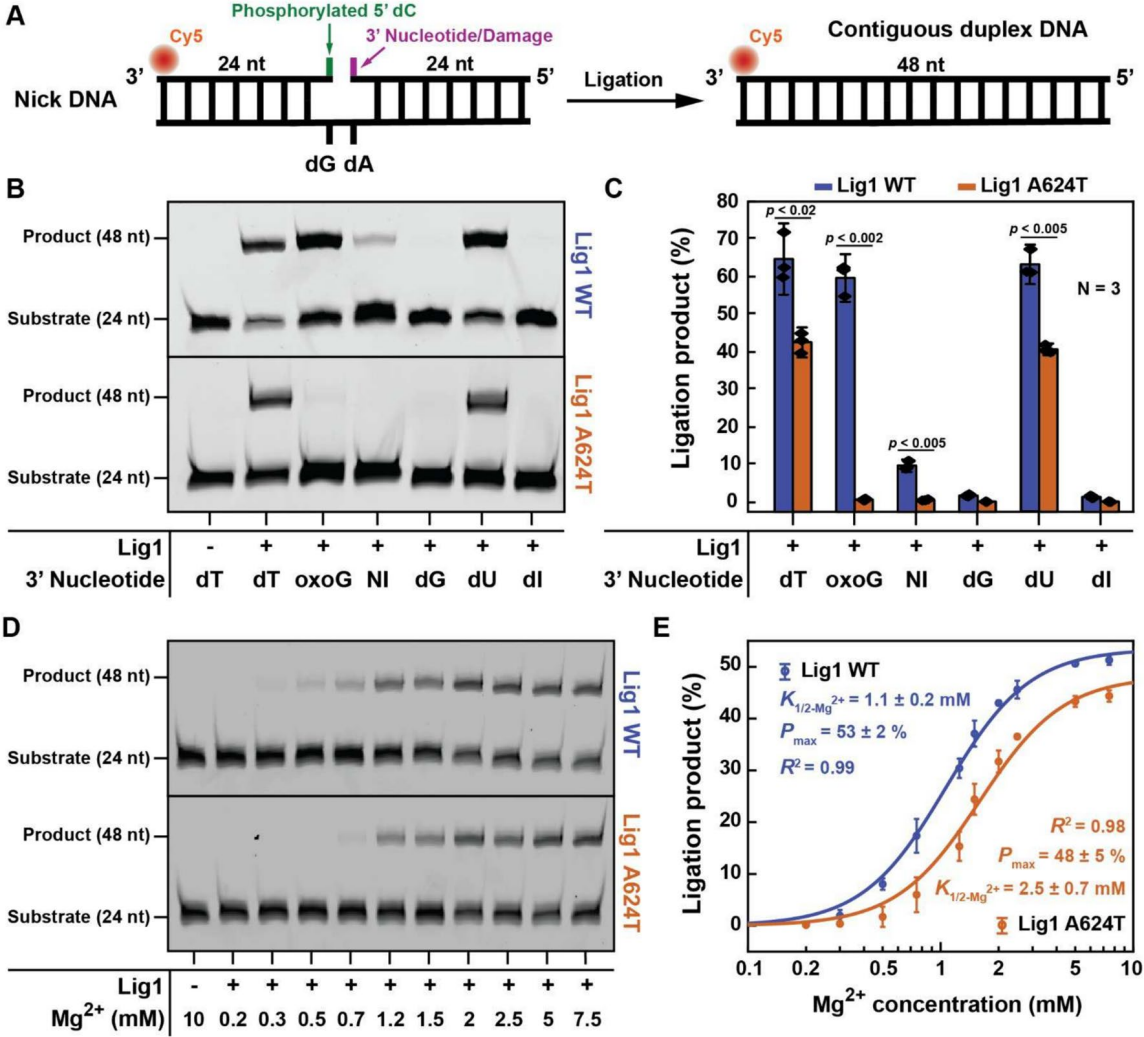
Reduced catalytic activity and enhanced replication fidelity in the A624T mutant protein. **A** Schematic representation of the ligation substrate and reaction. The nick substrate consists of three pre-annealed oligonucleotides: a 48nt template, a Cy5-labeled 24nt 5’arm phosphorylated at the 5’end to allow ligation, and a 24nt 3’arm containing either a perfectly-matched deoxyribonucleotide or a damage at the 3’end. **B** Denaturing Urea-PAGE gels showing the ligation product of nick substrates containing either a perfectly-matched deoxyribonucleotide (dT) or different damages at the 3’end of the 3’arm. **C** Quantification of ligation product as a percentage of total substrate from the ligation gels showed in panel B. The bar chart illustrates the mean (as bar height) and one standard deviation (as error bar) of three independent measurements. The p-values compare between the ligation generated by LIG1 WT versus A624T and are reported to the most significant digit. **D** Denaturing Urea-PAGE gels showing the ligation products for a nick substrate containing a perfectly-matched deoxyribonucleotide (dT) at the 3’end of the 3’arm at various Mg2 + concentrations for WT versus A624T mutant. **E** Quantification of ligation product as a percentage of total substrate from the ligation gels showed in panel D versus Mg2 + concentration. The data points illustrate the mean (as point location) and one standard deviation (as error bar) of three independent measurements. For both WT and A624T, the experimental data points were fitted to Hill equations with a fixed Hill coefficient of 2 to account for the two Mg2 + binding sites present in the enzyme

LIG1 activity was monitored using a ligation assay where a synthetic nick substrate is converted to a full dsDNA duplex upon ligation (Fig. 5A). The substrate was phosphorylated at the 5’end of the 5’arm of the nick to allow ligation. The same 5’arm was labeled at the 3’end with Cy5 fluorophore for reaction readout. The terminal 3’end nucleotide of the 3’arm of the substrate was varied to either a perfect-match natural nucleotide (dT), a mismatch (dG) or several chemically modified nucleotides (oxoG, NI, dU, and dI) (see Methods). Ligation fidelity should force LIG1 to abort activity on all substrates except for the perfect-match dT. Nevertheless, it was previously shown [19] that WT LIG1 tolerates 8-Oxoguanine at the 3’end of the 3’arm of the nick when base-paired to a natural deoxyadenosine (dA).

The first clear insight into the effects of the A624T mutation was offered by monitoring ligation of the cognate substrate that contains a perfect-match (dT paired to template dA) natural nucleotide at the 3’end of the 3’arm of the nick (Fig. 5B, C; dT). On this substrate, ligation by the mutant LIG1 was ~ 1.5-folds lower in yield as compared to WT. Furthermore, LIG1 A624T required higher Mg^2+^ concentration than LIG1 WT to achieve the same ligation yield in the same amount of reaction time (Fig. 5D). Quantitative characterization showed that LIG1 A624T has an ~ 2.5-folds weaker response to Mg^2+^ titration than the WT protein (Fig. 5E). Collectively, these results indicate that A624T causes a reduction in the catalytic activity of LIG1, which in turn may increase the genome-wide number of un-ligated nicks and cause genomic instability.

### A624T Leads to Enhanced Ligation Fidelity

The second insight into the mechanistic effects of the A624T mutation was offered by the investigation of ligation fidelity on sub-optimal substrates (Fig. 5B, C; oxoG, NI, dG, dU, and dI). Substrates containing 5-Nitroindole (NI; universal nucleobase), deoxyInosine (dI; nucleoside), and deoxyGuanosine (dG; nucleobase mismatch) at the 3’end of the 3’arm of the nick were well discriminated by both A624T and LIG1 WT and resulted in an almost complete lack of ligation. The substrate containing deoxyUridine (dU; nucleoside) was better discriminated by A624T as compared to the WT by ~ 1.5-folds. Nevertheless, this difference is within the range of the difference in catalytic activity between A624T and WT, thus it may not reflect a true difference in fidelity. The most striking observation is seen in the case of the substrate containing 8-Oxoguanine (oxoG; lesion) at the 3’end of the 3’arm of the nick. This substrate was ligated by WT with a yield similar to that of the cognate substrate (dT), yet it was almost completely aborted by LIG1 A624T (Fig. 5B). Accounting for the ~ 1.5-folds difference in catalytic efficiency of the two enzyme variants, quantification analysis shows that A624T has > 50-folds improved fidelity against 3’end oxoG as compared to the WT protein.

### Molecular Dynamic Simulation Reveals Allosteric Effects in the Mutant Protein

**Fig. 6:**
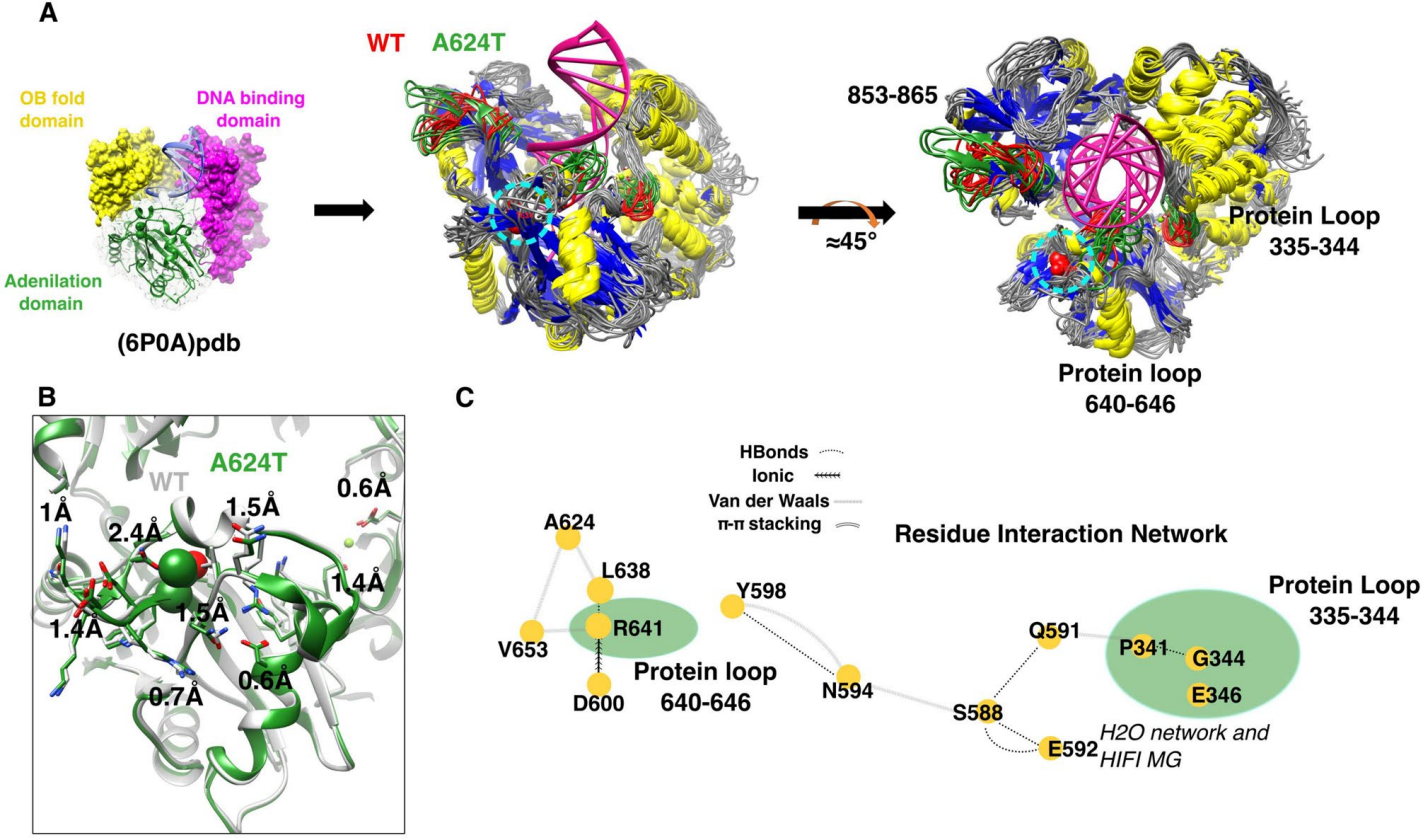
Effects of the A624T mutation on protein loop dynamics. **A** Ligase 1 has three domains, the A624T mutation is in adenylation domain (green spherical representation). The conformational display of the protein exhibited during MDs is shown for WT LIG1 and A624T. The superpositions of protein structure dynamics are shown to highlight the three protein loops that exhibited different molecular dynamics in red (WT) and green (A624T). Protein loop 335–344 is directly involved in stabilizing HIFI Mg in the DNA-binding domain, whereas protein loop 640–646 is involved in DNA stabilization. Alpha helices, beta sheets and the remaining protein loops are shown in yellow, blue and gray respectively, and DNA in magenta. **B** The atomic coordination adjustment (Å) undergone in the vicinity of the A624T mutation is shown. The mutated amino acid is shown by the spherical representation, the rest of the side chain atoms are shown in the stick representation with oxygen and nitrogen atoms in red and blue respectively. **C** The residue interaction network for WT ligase 1 was created to show the physical contacts connecting the mutation site to the affected protein loops through the LIG1 domains. The 335–344 and 640–644 loops of the protein are highlighted with green ovoids

As a corollary to our substrate studies, we conducted molecular dynamic (MD) simulations to explore how A624T might perturb the protein-substrate complex. Using coarse-grained force field, we identified clear localized differences in three specific protein loops between WT and mutant (335–344, 640–646 and 853–865; Fig. 6A). All these loops were oriented towards the dsDNA. The 640–646 loop is the main adenylation domain, and the 335–344 loop is used by the DNA-binding domain to form the H_2_O network that stabilizes the high-fidelity (HIFI) Mg [19].

The relative bulking of the mutant threonine side chain, nearly doubling the size of the wildtype alanine, was shown to add a polar moiety oriented towards the back of an alpha helix, leading to the secondary structure of the protein-DNA interface (Fig. 6B). A624T also affected the packing of the side chains and the backbone of the adenylation domain core in the vicinity. The adjustment needed to avoid atomic clashes appeared to be changing the atoms’ coordination in a range of 0.6 Å-2.4 Å; a range that could alter not only the H-bond network, but also other non-covalent interactions involved in stabilizing protein structure and function.

Interestingly, position 624 is not part of the protein-DNA contact interface, the protein loop that stabilizes DNA binding (640–646), nor is it close to the HIFI Mg-stabilizing region in the DNA binding domain. Therefore, we decided to perform a Residue Interaction Network (RIN) analysis to study the mutation’s allosteric effect. The RIN analysis of WT LIG1 showed no direct connection between position A624 (Fig. 6C) and the 335–344 protein loop, or with E592, both of which are involved in HIFI Mg stabilization. However, after introducing the mutation, the surrounding amino acids readjust to a more stable and favorable configuration that seems to alter the RIN (Fig. 6B).

**Fig. 7:**
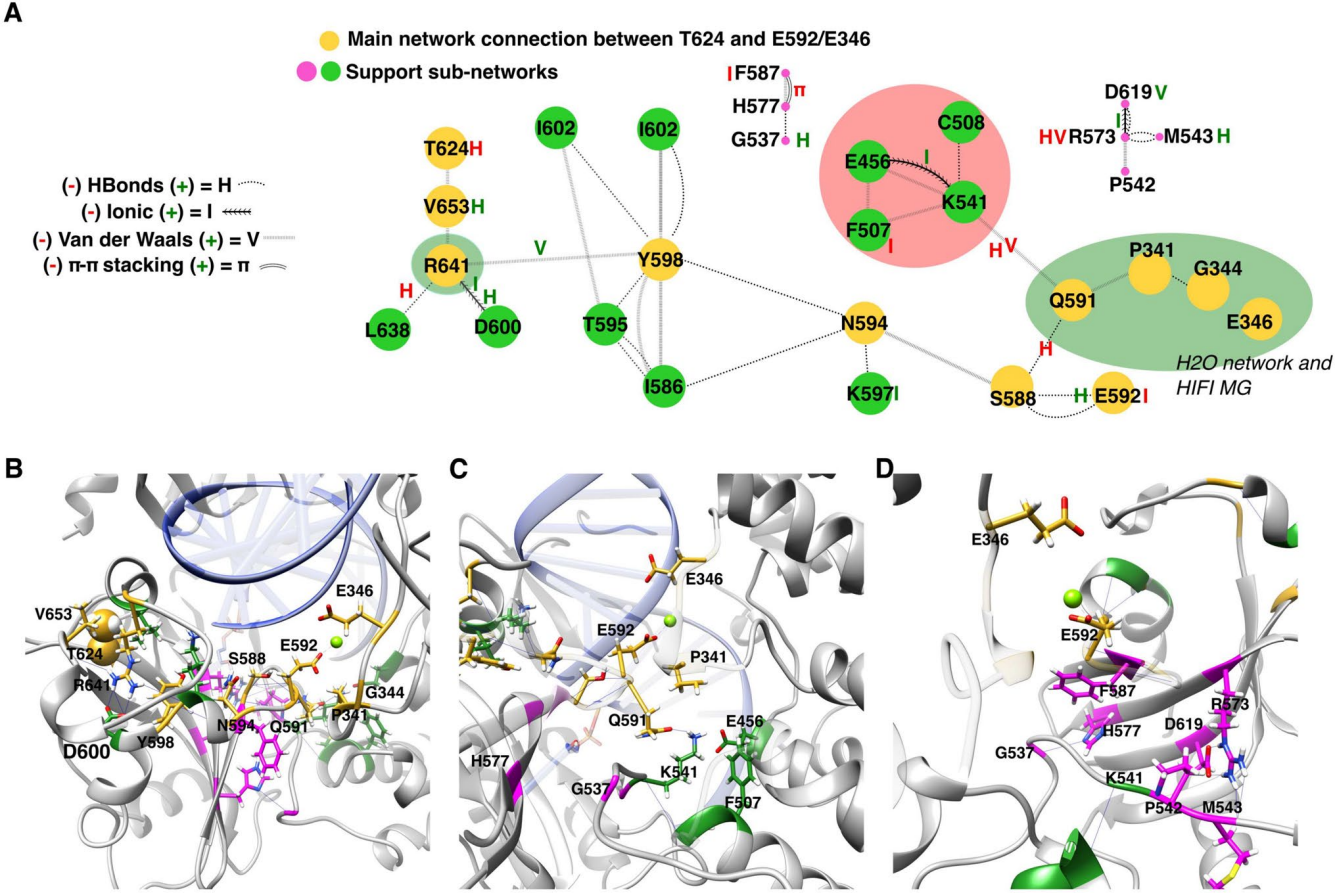
Effects of the A624T mutation on the connectivity network of LIG1. **A** Comparison of Residue Interaction Network (RIN) analysis of WT and A624T proteins is shown. Connectivity changes are illustrated with red or green letters, for loss and gain of a physical interaction. The residue network connecting the T624 mutation to protein loops 335–344 and 640–646 is highlighted with yellow circles (amino acids in both protein loops are identified with green ovoids). Two supporting networks stabilizing the main connectivity network are presented as magenta and green nodes. **B** 3D amino acid coordination of the LIG1 WT connectivity network (color-coded as in panel A) is presented. The mutation at 624 is shown as spherical atoms and hydrogen bonds are shown as thin black lines. **C** Stabilization of Mg (lime green) by E592 is shown, as well as the involvement of the supporting sub-network K541, E456 and F507 (dark green). Also shown is the stabilization of Q591 by H-bonds that allow the orientation of E592 toward the Mg HIFI atom. **D** The effect of the A624T mutation on E592 and HIFI Mg seems to be related also to the loss of π-π stacking interactions (F587 and H577), H-bonds and Van Der Walls connections (R573) that stabilize the protein loop where K541 is located. This therefore does not allow the stabilization of E592 through its backbone connection with Q591

We next compared the conformational sampling obtained during MDs and RIN analysis to determine which connections were lost or gained during the simulation (Fig. 7A). While the WT kept E592 well connected to the protein loop 335–344 (in charge of HIFI Mg stabilization) via a molecular bridge of S588 and Q591, the mutant favored their disconnection, causing an increase of their molecular dynamics and affecting the range of their displacement towards the protein-dsDNA contact interface (Fig. 6A, in green). This disconnection of the protein loop 335–344 is due to a decrease in the constraints applied by the sub-networks identified by RIN (highlighted in green and magenta, Fig. 7B-D). While the A624T mutation expands its effects to the core of the adenylation domain through amino acids R641 and Y598 towards S588, there appears to be a decrease in π-π interactions over the F587-H577 sub-networks and a loss of Van der Waals interactions and H-bonding at E573 that no longer constrain the orientation of the loop where K541 is located (Fig. 7D). This amino acid (K541) is important for the proper location of Q591 and the proper conformation of E592 towards Mg (Fig. 7C).

The MD results are in agreement with previous reports, where alanine scanning revealed that alteration of E346 (HIFI Mg stabilization section of the DNA binding domain) or E592 could reduce the kinetic constants of LIG1 by up to three times [19]. A series of rapid quenching experiments showed that WT and a E346A/E592A double mutant exhibited similar performance activities with a normal DNA substrate, proving that these two mutations did not disrupt catalysis. Collectively, our data suggest an allosteric effect of the A624T mutation on the protein loops involved with HIFI Mg as well as the adenylation domain’s DNA binding (loop 640–646).

## Discussion

LIG1 deficiency represents an extremely rare form of inborn error of immunity characterized by diverse clinical manifestations. Including this study, only eight cases have been documented since its initial description 32 years ago [10, 12, 14], as summarized in Table 1. Clinical onset typically occurs from birth to two years of age. Affected patients may present with SCID, Omenn-like syndrome, or other combined immunodeficiency phenotypes. Severe viral respiratory and gastrointestinal infections are common, along with severe macrocytic anemia which is likely related to impaired DNA synthesis in hematopoietic stem cells. Immunologically, patients often exhibit severe T and B cell lymphopenias, expansion of γδ-T cells, and panhypogammaglobulinemia.

Management strategies include immunoglobulin therapy, prophylactic antibiotics, and HSCT. To date four patients have undergone HSCT, with three receiving reduced-intensity conditioning due to concerns about chemotherapy-induced toxicity in DNA repair defects [14, 20]. One of these patients achieved 100% donor lymphoid and myeloid engraftment, curing both the immune defect and anemia. The other two cases showed mixed donor cell engraftment, resulting in improved T- but poor B-cell function, and continued to require blood transfusions. Our case is the first reported instance of HSCT without conditioning, achieving 87% lymphoid and 4% myeloid cell engraftment. The patient showed improved T cell function but exhibited poor B cell engraftment and mild macrocytic anemia, yet did not require blood transfusions. Further data are urgently needed to establish the optimal conditioning protocol for LIG1 deficiency, ensuring high donor cell engraftment to fully correct the disease phenotype.

Here we describe the genetic and molecular findings associated with a novel mutation in the *LIG1* gene, which leads to an A624T residue change that affects the protein’s interaction with the catalytic Mg^2+^ ion. Cognate substrate ligation experiments indicated reduced catalytic activity in the mutant, which may result in increased unligated nicks throughout the genome and concomitant genomic instability. Most likely this reduced efficiency is affecting ligation during the maturation of Okazaki fragments, where LIG1 must also compete with other proteins for successful ligation [5, 21], thus amplifying its deleterious effect. Mechanistically, we show that the mutant’s reduced ligation yield is directly due to its lower Mg^2+^ affinity. Conversely, our data also show a near-complete inability of the mutant to process oxoG, indicating a > 50-fold increased fidelity against this lesion.

This higher fidelity against 3’ end oxoG, also observed in LIG1 mutants R641L and R771W [13], might at first glance appear to be a gain of function rather than a defect, since oxoG should be excluded from the final mature genome due to its connection to mutagenesis and carcinogenesis. 8-oxoG is naturally generated by radical oxygen species and represents the most abundant and prevalent DNA lesion [22, 23]. 8oxoG/C pairs are corrected by the specific glycosylase OGG1 during or post-DNA replication. On the other hand, 8oxoG/A pairs are primarily removed post-replication by the base excision repair (BER) pathway [23]. Therefore, achieving timely DNA replication may necessitate temporarily accepting the incorporation of 8-oxoG into the ligated genome, only to repair it later through BER, rather than leaving behind numerous nicks through abortive ligation. In this context, the higher fidelity of A624T would produce increased genomic fragmentation, especially in the presence of high oxidative stress.

Our MD and RINS analysis revealed that doubling of the side chain volume, and the polar effect introduced by the A624T hydroxyl group, reorganizes the molecular packing in the core of the adenylation domain. Such an event may provoke an uncoupling of the stabilization residues that ensure the atomic coordination of glutamic acid 592 and the 335–344 protein loop that plays a decisive role in stabilizing the high-fidelity magnesium.

While our study corroborates earlier findings that show enhanced fidelity as a mechanism underlying LIG1 dysfunction, it also highlights the lack of a clear genotype–phenotype correlation, though this is partially confounded by the limited number of patients reported to date. However, the literature does show certain compound heterozygous patients who had one allele coding for a truncated product, and the other coding for a protein with defective oxoG processing, who had a relatively mild clinical course. These individuals experienced an age of onset at 2 years, had defects in antibody production and a CVID-like picture, and were managed via immunoglobulin treatment. On the other hand there are patients homozygous for alleles encoding defective oxoG processing, wherein the age of onset is in the 2–4 month range and the clinical management generally necessitates HSCT transplantation [12, 13]. Our patient, with a more SCID-like presentation, falls towards the severe end of the phenotypic spectrum. This phenotypic disparity highlights the central role of each patient’s genetic background in determining clinical severity and indicates that the exact molecular nature of the LIG1 defect is not sufficient to predict the expected clinical course for the patient.

The earliest reported LIG1-deficient fibroblast cell line, derived from the initial LIG1 patient, revealed an increased sensitivity to ionizing radiation in line with our patient findings [24]. These SV40-transformed cells (46BR.1G1) demonstrated 3–5% normal LIG1 activity and delayed maturation of replication intermediates [9, 25], resulting in the buildup of both SSBs and DSBs [26]. The latter likely stemmed from the cells’ attempt to handle SSBs that are a by-product of this defective maturation. Unrepaired SSBs cause complex DSBs [27], as seen by our patient cells’ elevated baseline gamma-H2AX levels.

In 46BR.1G1, gamma-H2AX foci form in the S phase and persist through metaphase and telophase, indicating that DNA damage does not prevent cell division [26]. The number of foci then diminishes in G1, possibly due to compensatory repair by LIG3 and LIG4. This damage does not appear to impede the cell cycle, since the proportion of cells in each cell cycle phase remains almost constant following complementation with a WT LIG1 vector [26]. In contrast, cell cycle analysis of our patient fibroblasts (without treatment) shows a significantly higher percentage of G1 cells than in controls or 46BR.1G1 data. This discrepancy between the two LIG1-deficient fibroblast cell lines may be due to the use of primary cells versus a simian virus-transformed cell line.

Two main checkpoints assess DNA integrity throughout the cell cycle. ATM/Chk2 is located prior to S phase entry, and searches for the presence of DSBs [28]. The general G1 block in our untreated patient cells suggests that this checkpoint is active. The fact that so few cells are in G2 implies that ATR/Chk1, the checkpoint that blocks entrance into mitosis in the presence of SSBs, is not monitoring the damage caused by faulty replication. However, exposure to ionizing radiation causes a significant and prolonged G2 block. Ionizing radiation produces roughly 50-fold more SSBs than DSBs [29]. This implies that the ATR/Chk1 checkpoint is discriminating between SSBs caused by radiation damage and those caused by replication defects.

Taken together, our results show that the LIG1 A624T defect stems from a combination of reduced ligation efficiency on cognate substrates, and excessive abortive ligation on non-cognate substrates which contain lesions that should be tolerated, at least temporarily, until other pathways can intervene to correct them. The acuteness of our patient’s clinical presentation highlights the presence of these two mechanistic factors, both impacting LIG1 function in disparate ways and stemming from a single mutation.

### Supplementary Information

Below is the link to the electronic supplementary material.

Supplementary file1 (PDF 707 KB)

## Data Availability

The datasets presented here are protected under local data privacy laws, and cannot be made publicly available. Requests to access the datasets should be directed to the corresponding author (amalazami@kfshrc.edu.sa).
