# Supplementary material for: Severe Combined Immunodeficiency from a Homozygous DNA Ligase 1 Mutant with Reduced Catalytic Activity but Increased Ligation Fidelity
Source: J Clin Immunol. 2024 Jun 19;44(7):151. doi: 10.1007/s10875-024-01754-1 (PMC11186889; doi:10.1007/s10875-024-01754-1)
Supplement: Supplementary file 1 — Supplementary file1 (PDF 707 KB) [file 10875_2024_1754_MOESM1_ESM.pdf]

Severe Combined Immunodeficiency from a Homozygous DNA Ligase 1 Mutant with Reduced Catalytic Activity but Increased Ligation Fidelity

Supplementary Methods

Study approval

All research involving patient materials was approved by the King Faisal Specialist Hospital & Research Centre (KFSHRC) institutional review board, RAC # 2080 025. Informed consent was obtained from the individuals themselves (for adults) or their legal guardians (for minors). Clinical and family history was acquired along with blood samples from the family members who were available for recruitment (Fig 1A), as well as skin biopsy from the patient only.

Whole exome and sanger sequencing

A rapid whole exome sequencing (Flash WES) protocol was utilized as described elsewhere [1]. Briefly, rapid DNA extraction was carried out with the PureLink Genomic DNA kit (Thermo Fisher). The Ion Torrent AmpliSeq WES protocol was then followed for library preparation, emulsion PCR, and enrichment and sequencing steps. Reads were mapped against the reference genome (hg19) by BWA. SNPs and Indels were detected by Samtools and annotated via ANNOVAR [2]. For filtering of the resultant data, only homozygous variants that were exonic or splite site in nature (using UCSC Genes as our reference standard) were considered. Those with allele frequencies > 0.002 in publically available repositories (or our own in-house database) were also removed. Surviving variants were further refined based on their physical location within autozygome regions exclusive to the patient, along with predicted

pathogenicity using the SIFT and Polyphen-2 online programs. Variant counts at each step of our filtering scheme are presented in Figure 1B. For Sanger sequencing, genomic DNA was obtained from whole blood samples, and sequenced using the BigDye Terminator. Sequencing results were analysed by SeqMan Pro (DNA Star Inc.) for mutation verification and to confirm segregation.

Genomewide high-density genotyping

Genomewide SNP genotyping and homozygosity mapping was performed via the AxiomGWH SNP Chip platform (Affymetrix). The AutoSNPa v4 and Multi-Ideogram programs were used to determine and visualize the autozygome from the genotype data. For calling autozygous intervals we implemented a size limit of 2.0 Mb. This allowed us to utilize autozygosity for purposes of WES filtering, as indicated above, without being encumbered by short identical-by-state regions of homozygosity which are abundant in populations with high consanguinity levels.

Immunophenotyping

Frozen PBMCs were thawed, washed then left to rest for 2 hours in complete medium. Cells were counted and suspended at 10^6 cells per ml in FACS buffer (2% fetal calf serum in PBS), then stained and analyzed on an LSR II flow cytometer (Becton Dickinson, Mountain View, CA, USA). All antibodies were purchased from BD Biosciences (San Jose, CA, USA). T cell analysis involved antibodies against CD3-Amcyan, CD4-PerCP-Cy5.5, CD8-PE-Cy7, CD45RO-FITC and CD27-PE. This allowed identification of the naïve ($CD45RO^{-}CD27^{+}$), central memory ($CD45RO^{+}CD27^{+}$), effector memory ($CD45RO^{+}CD27^{-}$) and effector ($CD45RO^{-}CD27^{-}$) compartments from gated $CD3^{+}/CD4^{+}$ or $CD3^{+}/CD8^{+}$ T cells. NK cells

were identified as $CD3^-$ cells that were positive for CD16-APC and CD56-PE staining. For B cells, antibodies against CD19-PerCP-Cy5.5, CD27PE-Cy7, CD38-APC, CD24-PE, IgD-FITC and IgM-APC-Cy7 were used to identify naïve ($CD27^-IgD^+$), class-switched memory ($CD27^+IgD^-$), transitional ($CD24^{high}CD38^{high}$) and plasmablast ($CD24^-CD38^+$) fractions from gated live ($DAPI^-$) $CD19^+$ cells.

Fibroblast assays

Fibroblast cells were cultured from the patient's skin biopsy. To gauge proliferation rate, fibroblasts were seeded at low (1×10^4) or high (5×10^4) densities per 6 cm plate. At specific intervals cells were then analyzed using crystal violet (Sigma) to determine change in biomass as a proxy for cellular growth. For the survival assay, patient and control fibroblast cells were seeded at equal densities (7.5×10^4). Next day some cells were processed using crystal violet to obtain a baseline reading (day 0), while the remainder were subjected to H_2O_2 (200 µM) for varying time periods then washed and allowed to recover for 72 hours before also being processed. Crystal violet readings from 72 hours post-treatment were then normalized to their day 0 counterpart, to determine how well each cell line had recovered from H_2O_2 treatment. For viability, fibroblast cell lines seeded in 6 cm plates (7.5×10^4 per well) were allowed to grow for 24 hours then either treated, or not, with H_2O_2 (200 µM) for 30 minutes. Following a 48 hour recovery period, 4′,6-diamidino-2-phenylindole (DAPI) was added and cells were examined by flow cytometry to determine the DAPI-negative (viable) percentages.

Exogenous protein expression

The *LIG1* open reading frame, both WT and mutant forms, were subcloned into a pcDNA3.1+/C-(K)-DYK vector backbone containing a C-terminal FLAG tag, to generate

expression vectors (GenScript). Human embryonic kidney HEK293 cells (ATCC) were grown in Dulbecco's Modified Eagle Medium (Invitrogen) supplemented with 10% fetal calf serum. Next day, at ~70% confluence, cells were transfected with Lipofectamine 3000 (Invitrogen) using standard protocols. Proteins were extracted 24 hours later.

Immunoblotting

Lysates from fibroblast or HEK293 cell lines were obtained using RIPA buffer (Sigma-Aldrich) supplemented with a protease and phosphatase inhibitor cocktail mix (Thermo Fisher). Following SDS-PAGE electrophoresis (National Diagnostics), samples were transferred onto a PVDF membrane (GE Healthcare). HRP-conjugated secondary antibodies were detected by SuperSignal WestPico Chemiluminescent (Thermo Scientific). The following primary antibodies were used: rabbit anti-gamma H2AX (pSer139, NB100-384, Novus Biologicals), mouse anti-PCNA (sc-56, Santa Cruz Biotechnology), mouse anti-beta Actin (ab8226, Abcam), mouse anti-FLAG (F1804, Sigma-Aldrich), and rabbit anti-GAPDH (2118, Cell Signaling Technology). Densitometry readings for quantification of band intensities were obtained using ImageJ (v. 1.46r) software.

Radiation sensitivity assay

To gauge sensitivity to ionizing radiation, patient and control fibroblast cells were trypsinized and seeded onto sterile coverslips coated with poly-L-lysine (Sigma-Aldrich), and left to grow overnight in complete medium. Next day, cells were (or were not) exposed to 4 Gy radiation using an X-RAD320 system (Precision X-ray Inc.), then allowed to recover for the indicated time points. Cells were then fixed with pre-warmed 3.6% formaldehyde and permeabilized with 0.5% Triton X-100 (Sigma-Aldrich). Following a 1 hour room temperature incubation

with anti-gamma H2AX primary antibody (pSer139, NB100-384, Novus Biologicals), cells were washed then further incubated for another hour with a FITC-labelled secondary antibody (F-2765, Thermo Fisher). Coverslips were mounted in Hardset Vectashield containing DAPI (Vector Labs) and observed under a fluorescent microscope (Zeiss Axio Imager.Z2). On average 45 cells were scored from each coverslip for each cell line and recovery period, with each experiment performed in duplicate.

Cell cycle analysis

After overnight culturing in complete medium, patient and control fibroblast cells were exposed to 4 Gy ionizing radiation (as above) then allowed to recover. At the indicated time points cells were trypsinized, washed, then fixed in 70% ice-cold ethanol till batches were ready for analysis. Cells were treated with Ribonuclease A (Sigma-Aldrich), stained with propidium iodide (Calbiochem) then analyzed on a BD FACSCalibur with a minimum of 10000 events per sample. Demarcation of cell cycle phases was performed using BD CellQuest software.

Protein expression and purification

Human LIG1 WT and A624T mutant proteins were expressed and purified as described previously using a custom-made PCNA-Agarose column [3, 4]. Briefly, the cloned plasmids of *LIG1* WT and mutant DNA were transformed into BL21 (DE3) *E. coli cells separately*. The cells were grown in 2YT media at 37 °C to an OD_{600} of 0.7 and protein expression was induced with 0.2 mM IPTG and further incubated for 19 hours at 16 °C. The cells were collected by centrifugation and lysed by lysozyme and sonication. The debris was removed by centrifugation and the supernatant was loaded onto 10 ml PCNA-Agarose column followed by

a HiTrap Blue 5 ml (Cytiva) column. Finally, fractions containing LIG1 protein were concentrated and loaded onto HiLoad 16/600 Superdex 200 pg size exclusion column (Cytiva). LIG1 protein fractions were flash-frozen and stored at -80 °C.

DNA substrate annealing

All oligonucleotides were purchased from Integrated DNA Technologies (IDT) and were HPLC-purified by the manufacturer. Substrates were annealed by mixing all three oligos (template, 5'arm and 3'arm) at a 1:1:1 molar ratio in TE-100 buffer [50 mM Tris-HCl (pH 8.0), 1 mM ethylenediaminetetraacetic acid (EDTA), and 100 mM NaCl] and heated at 95 °C for 5 minutes followed by slow cooling to room temperature. Substrates were purified to >90% purity by running them on 10% non-denaturing polyacrylamide gel electrophoresis (PAGE) (Invitrogen). Finally, the substrates were aliquoted and stored at −20 °C. All oligos used for the ligation reactions are presented using IDT sequence codes in the table below:

Oligo name	IDT oligo sequence
Template	5'-GTGATGACGAGCAGTCCTAACTGGAAATCTAGCTCTGTGGAGTTCATG-3'
5'arm Cy5-labeled	5'-/5Phos/CCAGTTAGGACTGCTCGTCATCA/3Cy5Sp/-3'
3'arm Unmodified	5'-CATGAACTCCACAGAGCTAGATTT-3'
3'arm 8-Oxoguanine	5'-CATGAACTCCACAGAGCTAGATT/oxoG/-3'
3'arm 5-Nitroindole	5'-CATGAACTCCACAGAGCTAGATT/35NitInd/-3'
3'arm dG (Mismatch)	5'-CATGAACTCCACAGAGCTAGATTG-3'
3'arm deoxyUridine	5'-CATGAACTCCACAGAGCTAGATT/3deoxyU/-3'

3’arm deoxyInosine	5’-CATGAACTCCACAGAGCTAGATT/3deoxyI/-3’

DNA ligation assays

LIG1 WT and A624T mutant assays were performed in a buffer containing 50 mM HEPES-KOH pH 7.5, 5% (v/v) Glycerol, 1mM Dithiothreitol (DTT), 0.1 mg/ml bovine serum albumin (BSA), 100 mM KCl and 10 mM $MgCl_2$. In the case of the Mg^{2+}-titration experiments, the same buffer was used, but $MgCl_2$ was varied to the indicated concentration. For the ligation reactions, 10 nM nicked DNA substrate was incubated with 500 nM of LIG1, at 37°C for 30 seconds. The reactions were quenched by the addition of 40 mM EDTA. All reactions were incubated with Proteinase K at 50°C for 15 minutes and stopped by adding an equal volume of stop buffer (50 mM EDTA, 95% Formamide). DNA in the quenched reactions was denatured by heating at 95°C for 10 minutes and then immediately placed on ice. DNA reaction products were separated on 20% denaturing Urea-PAGE gels and visualized using Typhoon Trio (GE Healthcare).

Molecular dynamics simulations (MDs)

File 6P0A from the Protein Data Bank (PDB) was used for the *in silico* molecular binding analysis [5] with no further refinement due to the high resolution of the model (2.05Å). To compare the differences in the theoretical binding energies between WT and A624T, both protein models were minimized with the YASARA force field, and then the binding energies were estimated using the parameters of BindEnergy command implemented in Yasara Dynamics suite with AMBER14 [6]. During this process, the estimations of the binding energies were performed by comparing the DNA with respect to the rest of the atoms involved

in the simulation shell, including explicit solvent molecules. In order to avoid relying solely on an estimation system, the protein model was used again to calculate the changes in binding energy but using the alternative system of the Moldock scoring function in Molegro Virtual Docker [7].

MDs were performed to study the effect of the A624T mutation on the molecular behavior of LIG1. The 6P0A-based system with explicit solvent molecules was ~120,000 atoms. To minimize the required computational power for such a large size system, 2 replicates for each WT and mutant were performed. CABSFlex2.0 coarse-grained force field was used instead of all atoms MDs since it employs a multiscale strategy that allows for faster MDs with large systems [8, 9]. The standard configuration for coarse-grained simulation (CG-MDs) was used, but the number of cycles and the cycles between trajectories were extended to 100. Two rounds of MDs were performed for the WT and A624T models with randomized seed parameters each time. UCSF Chimera 1.16 and InkScape 1.0.1 were used for the preparation of the structural images [10].

Residue interaction network (RIN) analysis

RIN analysis was performed with the Cytoscape 3.9.1 suite [11] and the applications in RINalizer and Moduland2 [12-15], using the minimized versions of the WT and A624T proteins. The RIN for both models were calculated using the Network Generator of the RING server [16, 17]. Hydrogen bonding interactions, Van der Waals (VDW) forces, disulphide bridge formation, salt bridges, π-π stacking, and π-cation energies were considered to estimate residues connecting edges (network nodes); bond angles and energies were calculated using the default settings of the server. The RING 3.0 algorithm was applied on the snapshots of the

MD trajectory obtained from the MDs generated by CABSFlex2.0 [8] to perform a multi-step analysis of the changes experienced by the RIN during the MDs. A conformationally dependent map was created, grouping the contacts of the residue interactions by type. By coupling RIN analysis and the MD results, the specific physical interactions that were perturbed by the A624T mutation in the MDs were identified.

Supplementary References

1. Monies, D., et al., *Lessons Learned from Large-Scale, First-Tier Clinical Exome Sequencing in a Highly Consanguineous Population.* Am J Hum Genet, 2019. **104**(6): p. 1182-1201.
2. Saudi Mendeliome, G., *Comprehensive gene panels provide advantages over clinical exome sequencing for Mendelian diseases.* Genome Biol, 2015. **16**: p. 134.
3. Raducanu, V.S., et al., *Two chromatographic schemes for protein purification involving the biotin/avidin interaction under native conditions.* J Chromatogr A, 2020. **1621**: p. 461051.
4. Tehseen, M., et al., *Proliferating cell nuclear antigen-agarose column: A tag-free and tag-dependent tool for protein purification affinity chromatography.* J Chromatogr A, 2019. **1602**: p. 341-349.
5. Tumbale, P.P., et al., *Two-tiered enforcement of high-fidelity DNA ligation.* Nat Commun, 2019. **10**(1): p. 5431.
6. Maier, J.A., et al., *ff14SB: Improving the Accuracy of Protein Side Chain and Backbone Parameters from ff99SB.* J Chem Theory Comput, 2015. **11**(8): p. 3696-713.
7. Thomsen, R. and M.H. Christensen, *MolDock: a new technique for high-accuracy molecular docking.* J Med Chem, 2006. **49**(11): p. 3315-21.
8. Kuriata, A., et al., *CABS-flex 2.0: a web server for fast simulations of flexibility of protein structures.* Nucleic Acids Res, 2018. **46**(W1): p. W338-W343.
9. Kmiecik, S., et al., *Coarse-Grained Protein Models and Their Applications.* Chem Rev, 2016. **116**(14): p. 7898-936.
10. Pettersen, E.F., et al., *UCSF Chimera--a visualization system for exploratory research and analysis.* J Comput Chem, 2004. **25**(13): p. 1605-12.
11. Saito, R., et al., *A travel guide to Cytoscape plugins.* Nat Methods, 2012. **9**(11): p. 1069-76.
12. Doncheva, N.T., et al., *Topological analysis and interactive visualization of biological networks and protein structures.* Nat Protoc, 2012. **7**(4): p. 670-85.
13. Szalay-Beko, M., et al., *ModuLand plug-in for Cytoscape: determination of hierarchical layers of overlapping network modules and community centrality.* Bioinformatics, 2012. **28**(16): p. 2202-4.

14. Morris, J.H., et al., *structureViz: linking Cytoscape and UCSF Chimera.* Bioinformatics, 2007. **23**(17): p. 2345-7.

15. Morris, J.H., et al., *setsApp for Cytoscape: Set operations for Cytoscape Nodes and Edges.* F1000Res, 2014. **3**: p. 149.

16. Piovesan, D., G. Minervini, and S.C. Tosatto, *The RING 2.0 web server for high quality residue interaction networks.* Nucleic Acids Res, 2016. **44**(W1): p. W367-74.

17. Clementel, D., et al., *RING 3.0: fast generation of probabilistic residue interaction networks from structural ensembles.* Nucleic Acids Res, 2022. **50**(W1): p. W651-W656.

Figure S1

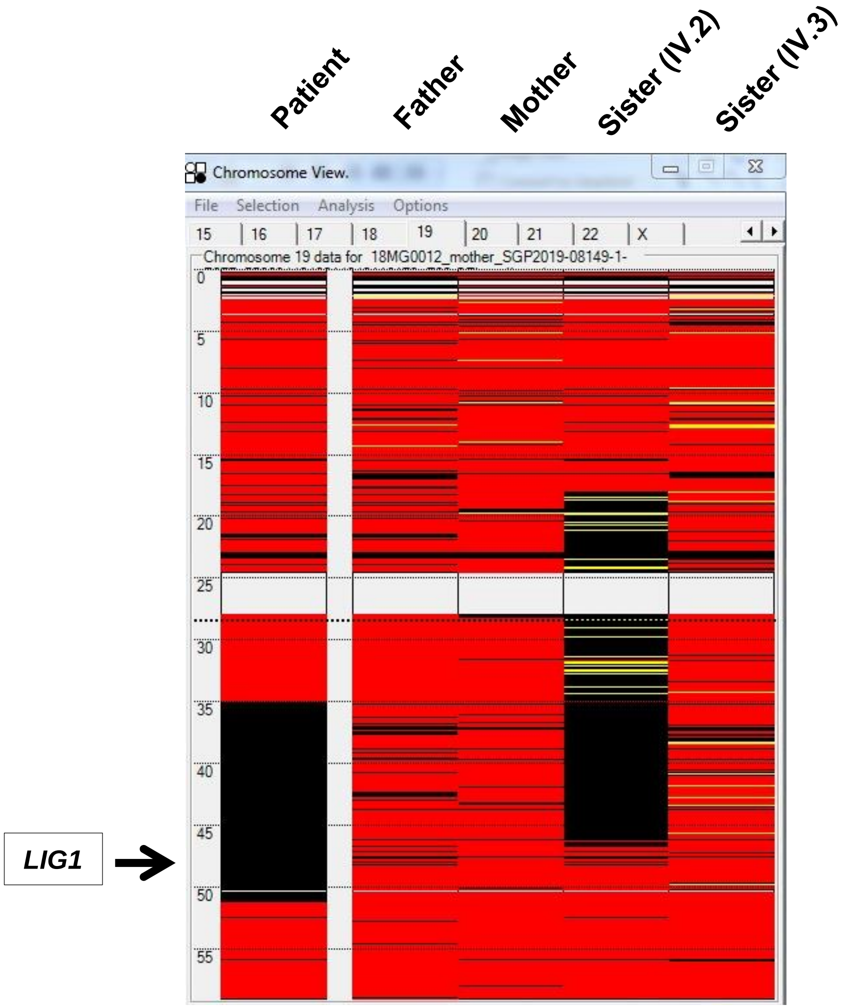

Figure S1. SNP genotyping confirms that only the patient is homozygous for the *LIG1* locus in this family. Output from AutoSNPa for chromosome 19, with heterozygous SNPs in red and homozygous ones in black. The heterozygous sister has a ~28 Mb autozygous stretch on this chromosome which does not include *LIG1*. The sisters are numbered as per Fig 1A.

Figure S2 (next page). Patient fibroblasts show cell cycle defects, in the presence and absence of induced DNA damage. (A) Representative histograms of propidium iodide staining of the patient and one control for every time point. Cells were exposed to 4 Gy radiation then left to recover for the indicated number of hours (0 = no treatment). Patient cells sustain a block in the G2 phase post-treatment and are unable to efficiently progress through mitosis, unlike control cells. A total of two independent experiments were conducted against two different controls each time (for a total of four controls), each performed in duplicate. (B) Bar graph visualization of the data from “A”, indicating the percentage of cells observed in each cell cycle phase per recovery time point. At T0 a significant majority of the patient cells are clustered in G1, with extremely few cells in G2. This changes into a G2 block post-radiation, where cells remain in significant numbers even at 72 hours recovery. Asterisks indicate significance levels (*$p < 0.05$, **$p < 0.01$; unpaired Student’s t-test). Error bars indicate SEMs.

Figure S2

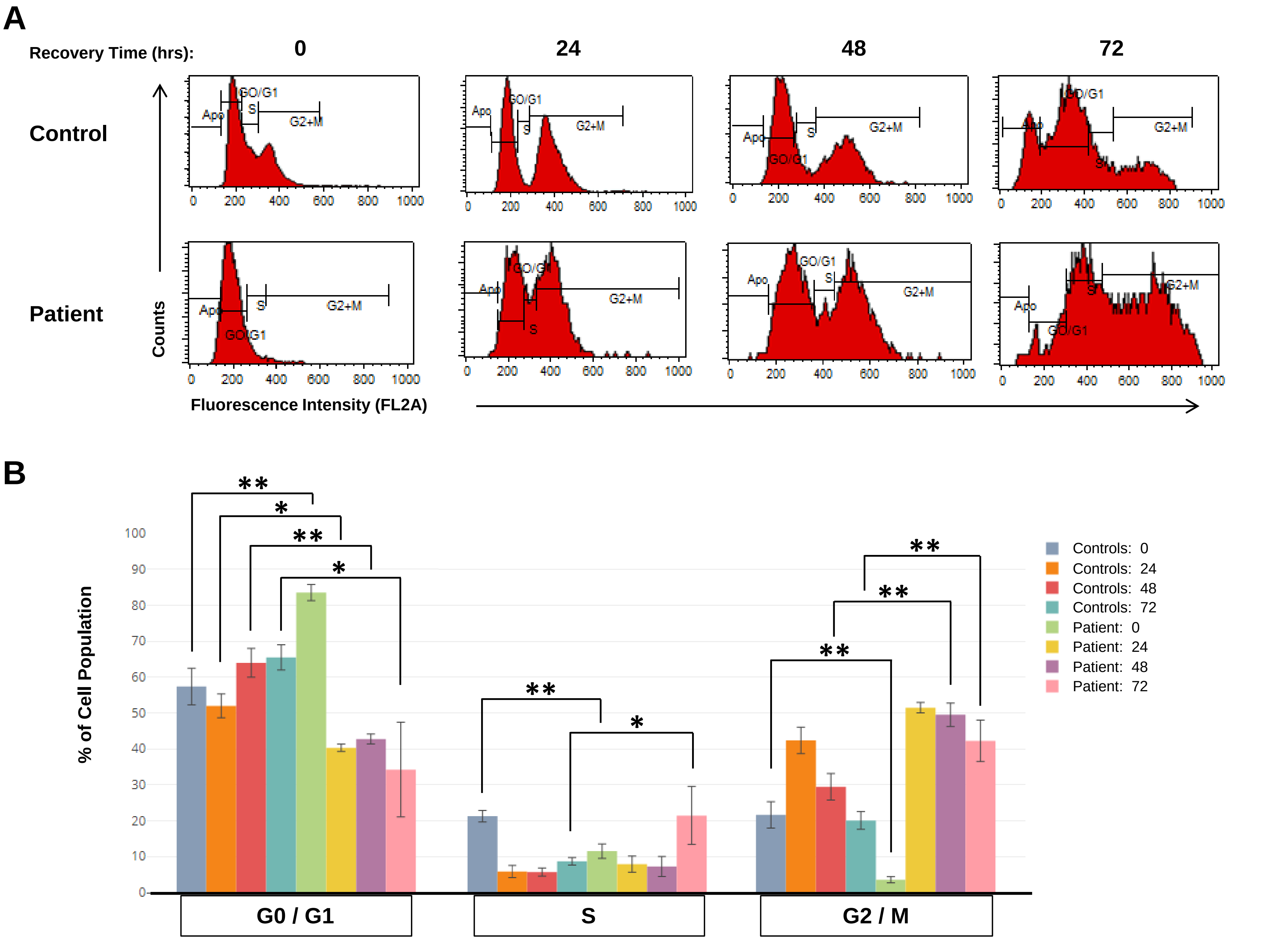
A
Recovery Time (hrs):
0
24
48
72
Control
Patient
Counts
Apo
GO/G1
S
G2+M
0
200
400
600
800
1000
Fluorescence Intensity (FL2A)
B
% of Cell Population
100
90
80
70
60
50
40
30
20
10
0
G0 / G1
S
G2 / M
Controls: 0
Controls: 24
Controls: 48
Controls: 72
Patient: 0
Patient: 24
Patient: 48
Patient: 72
**
*
